# Diethyl 4-hy­droxy-4-methyl-6-oxo-2-phenyl­cyclo­hexane-1,3-dicarboxyl­ate

**DOI:** 10.1107/S1600536810025018

**Published:** 2010-07-03

**Authors:** Hoong-Kun Fun, Madhukar Hemamalini, Mahesh Padaki, Arun M Isloor

**Affiliations:** a X-ray Crystallography Unit, School of Physics, Universiti Sains Malaysia, 11800 USM, Penang, Malaysia; b Organic Chemistry Division, Department of Chemistry, National Institute of Technology, Karnataka, Surathkal, Mangalore 575 025, India

## Abstract

In the title mol­ecule, C_19_H_24_O_6_, the cyclo­hexa­none ring adopts a chair conformation. The dihedral angle between the phenyl ring and the best plane through the six atoms of the cyclo­hexa­none ring is 89.68 (7)°. In the crystal structure, mol­ecules are linked *via* pairs of inter­molecular O—H⋯O hydrogen bonds into centrosymmetric dimers and these dimers are connected by C—H⋯O inter­actions into columns down the *a* axis.

## Related literature

For the applications of phenyl­cylcohexane, see: Adly *et al.* (2004 ▶); Pohl *et al.* (1977 ▶); Chu *et al.* (2005 ▶). For ring conformations, see: Cremer & Pople (1975 ▶). For the stability of the temperature controller used in the data collection, see: Cosier & Glazer (1986 ▶).
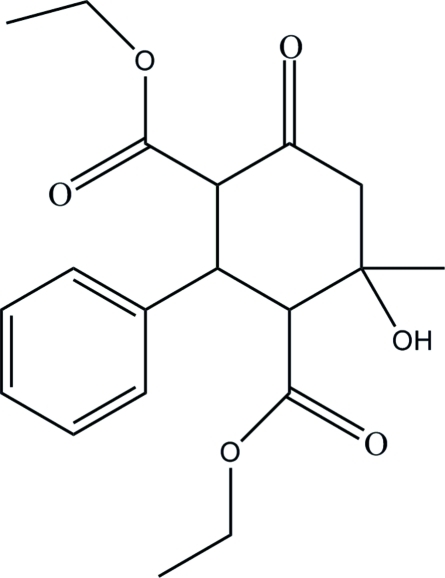


## Experimental

### 

#### Crystal data

C_19_H_24_O_6_
*M* *_r_* = 348.38
Monoclinic, 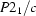

*a* = 5.792 (2) Å
*b* = 15.766 (6) Å
*c* = 20.031 (7) Å
β = 98.531 (10)°
*V* = 1808.9 (11) Å^3^
*Z* = 4
Mo *K*α radiation
μ = 0.10 mm^−1^
*T* = 100 K
0.40 × 0.10 × 0.06 mm

#### Data collection

Bruker APEXII DUO CCD area-detector diffractometer
Absorption correction: multi-scan (*SADABS*; Bruker, 2009 ▶) *T*_min_ = 0.963, *T*_max_ = 0.994
37474 measured reflections
5256 independent reflections
3644 reflections with *I* > 2σ(*I*)
*R*_int_ = 0.075

#### Refinement

*R*[*F* ^2^ > 2σ(*F* ^2^)] = 0.048
*wR*(*F* ^2^) = 0.122
*S* = 1.05
5256 reflections
233 parameters
H atoms treated by a mixture of independent and constrained refinement
Δρ_max_ = 0.33 e Å^−3^
Δρ_min_ = −0.23 e Å^−3^

### 

Data collection: *APEX2* (Bruker, 2009 ▶); cell refinement: *SAINT* (Bruker, 2009 ▶); data reduction: *SAINT*; program(s) used to solve structure: *SHELXTL* (Sheldrick, 2008 ▶); program(s) used to refine structure: *SHELXTL*; molecular graphics: *SHELXTL*; software used to prepare material for publication: *SHELXTL* and *PLATON* (Spek, 2009 ▶).

## Supplementary Material

Crystal structure: contains datablocks global, I. DOI: 10.1107/S1600536810025018/bt5276sup1.cif

Structure factors: contains datablocks I. DOI: 10.1107/S1600536810025018/bt5276Isup2.hkl

Additional supplementary materials:  crystallographic information; 3D view; checkCIF report

## Figures and Tables

**Table 1:** Hydrogen-bond geometry (Å, °)

*D*—H⋯*A*	*D*—H	H⋯*A*	*D*⋯*A*	*D*—H⋯*A*
O6—H1*O*6⋯O1^i^	1.01 (3)	2.05 (3)	2.9958 (18)	156 (2)
C1—H1*A*⋯O3^ii^	0.98	2.44	3.323 (2)	150
C8—H8*A*⋯O3^ii^	0.93	2.60	3.493 (2)	162
C12—H12*A*⋯O2^iii^	0.93	2.56	3.468 (2)	166
C19—H19*C*⋯O6^ii^	0.96	2.55	3.396 (2)	146

Symmetry codes: (i) 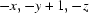; (ii) 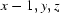; (iii) 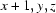.

**Fig. 1.**
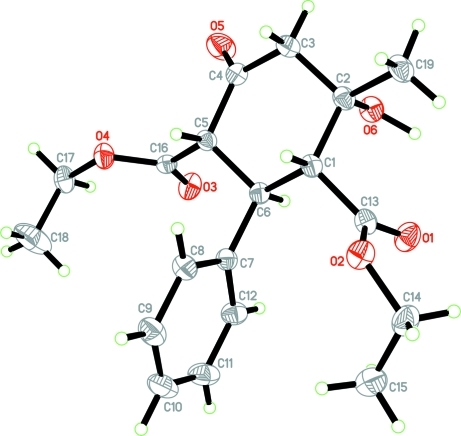
The asymmetric unit of the title compound, showing 50% probability displacement ellipsoids and the atom-numbering scheme.

**Fig. 2.**
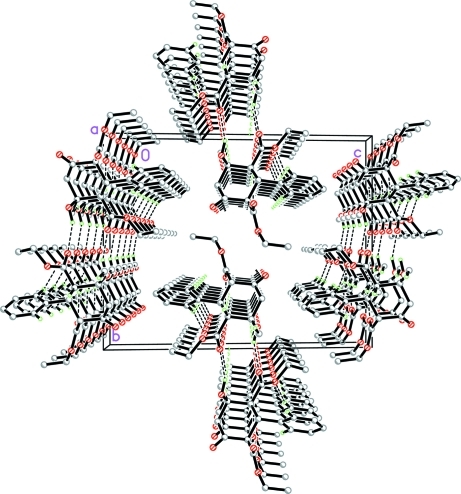
The crystal packing of the title compound, viewed down the a axis, showing the columns of dimers down the a-axis. H atoms not involved in the hydrogen bond interactions are omitted for clarity.

## supplementary crystallographic information

### Comment

Phenylcyclohexane is a highly valued chemical compound widely used as plasticizer in plastics, coatings and adhesive fields. It is also utilized as a penetrating agent. 5-Phenyl-cyclohexane-1,3-dione-4- carboxanilide is used as a stabilizer for double-base propellant and gives good results with the stability test (Adly *et al.*, 2004). Many substituted phenylcyclohexane derivatives have shown liquid crystal properties (Pohl *et al.*, 1977). Phenylcyclohexane derivatives are also biologically important. Linear pentapeptides (Penta-cis-Apc-DPhe-Arg-Trp-Gly-NH_2_) containing 1-amino-4-phenylcyclohexane-1-carboxylic acid (cis-Apc) and substituted Apc are potent hMC4R agonists and they are inactive or weakly active in hMC1R, hMC3R, and hMC5R agonist assays (Chu *et al.*, 2005). Keeping in view of the importance of the phenylcyclohexane derivatives, the title compound (I) was synthesized.

The molecular structure of (I), is shown in Fig. 1. The cyclohexanone ring adopts a chair conformation with puckering parameters (Cremer & Pople, 1975) Q = 0.5606 (14) Å, Θ = 172.71 (14)° and φ = 207.8 (11)°. The dihedral angle between the phenyl ring and the best plane through the six atoms of the cyclohexanone ring  is 89.68 (7)°.  In the crystal structure, Fig 2, adjacent molecules are linked via intermolecular O—H···O hydrogen bonds to centrosymmetric dimers. The dimers are connected by O6—H1O6···O1, C1—H1A···O3, C8—H8A···O3, C12—H12A···O2 and C19—H19C···O6 interactions (Table 1) into columns down the *a* axis.

### Experimental

Ethylacetoacetate (19.1 ml, 0.15 mol) and piperidine (9 ml) were dissolved in 150 ml of dry benzene. Then benzaldehyde (15.3 ml, 0.15 mol) was added drop-wise at room temperature over 20 min. The reaction mixture was slowly brought to boil and refluxed for 2 hours with constant stirring with periodic TLC monitoring. After cooling, organic layer was washed with cold aqueous 10 % sodium carbonate, water and 5 % acetic acid. Then the organic layer was dried and evaporated under reduced pressure and the crude product synthesis of 2-benzylidene-malonic acid diethyl ester was purified by crystallization from methanol. Separately ethylacetoacetate (8 mmol) was dissolved in ethanol (10 ml) and sodium acetate (6 mmol) was dissolved in water (2 ml) and then slowly added to the ethanol solution at room temperature. The resulting solution was stirred for 10 minutes and then added the 2-benzylidene-malonic acid diethyl ester (3.2 mmol) slot-wise. The reaction mixture was stirred for 24 hrs at room temperature. The solid formed was filtered and washed with water and recrystalised in ethanol. (yield 40 %, m.p 510–512 K).

### Refinement

The hydroxyl H atom was located from a difference Fourier map and refined freely. The remaining H atoms were positioned geometrically [C–H = 0.93–0.98 Å] and were refined using a riding model, with *U*_iso_(H) = 1.2*U*_eq_(C).

### Crystal data

**Table:**

C_19_H_24_O_6_	*F*(000) = 744
*M**_r_* = 348.38	*D*_x_ = 1.279 Mg m^−^^3^
Monoclinic, *P*2_1_/*c*	Mo *K*α radiation, λ = 0.71073 Å
Hall symbol: -P 2ybc	Cell parameters from 4276 reflections
*a* = 5.792 (2) Å	θ = 2.4–25.6°
*b* = 15.766 (6) Å	µ = 0.10 mm^−^^1^
*c* = 20.031 (7) Å	*T* = 100 K
β = 98.531 (10)°	Needle, colourless
*V* = 1808.9 (11) Å^3^	0.40 × 0.10 × 0.06 mm
*Z* = 4	

### Data collection

**Table:**

Bruker APEXII DUO CCD area-detector diffractometer	5256 independent reflections
Radiation source: fine-focus sealed tube	3644 reflections with *I* > 2σ(*I*)
graphite	*R*_int_ = 0.075
φ and ω scans	θ_max_ = 30.1°, θ_min_ = 2.1°
Absorption correction: multi-scan (*SADABS*; Bruker, 2009)	*h* = −8→8
*T*_min_ = 0.963, *T*_max_ = 0.994	*k* = −22→22
37474 measured reflections	*l* = −25→28

### Refinement

**Table:**

Refinement on *F*^2^	Primary atom site location: structure-invariant direct methods
Least-squares matrix: full	Secondary atom site location: difference Fourier map
*R*[*F*^2^ > 2σ(*F*^2^)] = 0.048	Hydrogen site location: inferred from neighbouring sites
*wR*(*F*^2^) = 0.122	H atoms treated by a mixture of independent and constrained refinement
*S* = 1.05	*w* = 1/[σ^2^(*F*_o_^2^) + (0.0484*P*)^2^ + 0.4474*P*] where *P* = (*F*_o_^2^ + 2*F*_c_^2^)/3
5256 reflections	(Δ/σ)_max_ < 0.001
233 parameters	Δρ_max_ = 0.33 e Å^−^^3^
0 restraints	Δρ_min_ = −0.23 e Å^−^^3^

### Special details

**Table:**

Experimental. The crystal was placed in the cold stream of an Oxford Cryosystems Cobra open-flow nitrogen cryostat (Cosier & Glazer, 1986) operating at 100.0 (1) K.
Geometry. All s.u.'s (except the s.u. in the dihedral angle between two l.s. planes) are estimated using the full covariance matrix. The cell s.u.'s are taken into account individually in the estimation of s.u.'s in distances, angles and torsion angles; correlations between s.u.'s in cell parameters are only used when they are defined by crystal symmetry. An approximate (isotropic) treatment of cell s.u.'s is used for estimating s.u.'s involving l.s. planes.
Refinement. Refinement of F^2^ against ALL reflections. The weighted R-factor wR and goodness of fit S are based on F^2^, conventional R-factors R are based on F, with F set to zero for negative F^2^. The threshold expression of F^2^ > 2σ(F^2^) is used only for calculating R-factors(gt) etc. and is not relevant to the choice of reflections for refinement. R-factors based on F^2^ are statistically about twice as large as those based on F, and R- factors based on ALL data will be even larger.

### Fractional atomic coordinates and isotropic or equivalent isotropic displacement parameters (Å^2^)

**Table:**

	*x*	*y*	*z*	*U*_iso_*/*U*_eq_	
O1	0.01230 (19)	0.45072 (6)	0.06556 (6)	0.0303 (2)	
O2	−0.26845 (17)	0.40000 (6)	0.12086 (5)	0.0264 (2)	
O3	0.47676 (16)	0.15467 (6)	0.04607 (6)	0.0272 (2)	
O4	0.24998 (17)	0.04265 (6)	0.06139 (5)	0.0249 (2)	
O5	0.1168 (2)	0.14395 (7)	−0.08748 (6)	0.0370 (3)	
O6	0.03208 (17)	0.36001 (7)	−0.05989 (5)	0.0249 (2)	
C1	−0.1446 (2)	0.31138 (8)	0.03806 (7)	0.0188 (3)	
H1A	−0.2822	0.2817	0.0494	0.023*	
C2	−0.1754 (2)	0.32386 (9)	−0.03951 (7)	0.0212 (3)	
C3	−0.1990 (2)	0.23718 (9)	−0.07382 (7)	0.0247 (3)	
H3A	−0.3412	0.2101	−0.0645	0.030*	
H3B	−0.2112	0.2447	−0.1223	0.030*	
C4	0.0059 (2)	0.18060 (9)	−0.04977 (7)	0.0232 (3)	
C5	0.0596 (2)	0.17099 (8)	0.02663 (7)	0.0183 (3)	
H5A	−0.0662	0.1379	0.0417	0.022*	
C6	0.0736 (2)	0.25755 (8)	0.06335 (7)	0.0182 (3)	
H6A	0.2101	0.2878	0.0517	0.022*	
C7	0.1109 (2)	0.24444 (8)	0.13937 (7)	0.0195 (3)	
C8	−0.0573 (2)	0.20405 (9)	0.17164 (7)	0.0240 (3)	
H8A	−0.1954	0.1852	0.1464	0.029*	
C9	−0.0195 (3)	0.19190 (10)	0.24098 (8)	0.0296 (3)	
H9A	−0.1327	0.1651	0.2619	0.036*	
C10	0.1857 (3)	0.21942 (11)	0.27934 (8)	0.0328 (4)	
H10A	0.2105	0.2113	0.3258	0.039*	
C11	0.3533 (3)	0.25901 (11)	0.24780 (8)	0.0326 (4)	
H11A	0.4917	0.2774	0.2733	0.039*	
C12	0.3165 (2)	0.27152 (9)	0.17833 (7)	0.0251 (3)	
H12A	0.4305	0.2983	0.1577	0.030*	
C13	−0.1227 (2)	0.39519 (9)	0.07513 (7)	0.0217 (3)	
C14	−0.2384 (3)	0.47213 (10)	0.16704 (8)	0.0304 (3)	
H14A	−0.3797	0.4802	0.1869	0.036*	
H14B	−0.2117	0.5231	0.1422	0.036*	
C15	−0.0354 (3)	0.45777 (11)	0.22215 (9)	0.0378 (4)	
H15A	−0.0292	0.5027	0.2547	0.057*	
H15B	0.1070	0.4569	0.2030	0.057*	
H15C	−0.0547	0.4045	0.2439	0.057*	
C16	0.2866 (2)	0.12288 (8)	0.04565 (7)	0.0191 (3)	
C17	0.4559 (3)	−0.00928 (10)	0.08439 (8)	0.0283 (3)	
H17A	0.5945	0.0184	0.0730	0.034*	
H17B	0.4407	−0.0640	0.0621	0.034*	
C18	0.4780 (4)	−0.02101 (15)	0.15860 (9)	0.0539 (6)	
H18A	0.6084	−0.0574	0.1735	0.081*	
H18B	0.3378	−0.0463	0.1697	0.081*	
H18C	0.5022	0.0331	0.1806	0.081*	
C19	−0.3862 (2)	0.37923 (10)	−0.06405 (8)	0.0275 (3)	
H19A	−0.4048	0.3838	−0.1123	0.041*	
H19B	−0.3631	0.4347	−0.0444	0.041*	
H19C	−0.5236	0.3541	−0.0509	0.041*	
H1O6	0.033 (5)	0.422 (2)	−0.0480 (14)	0.106 (10)*	

### Atomic displacement parameters (Å^2^)

**Table:**

	*U*^11^	*U*^22^	*U*^33^	*U*^12^	*U*^13^	*U*^23^
O1	0.0355 (6)	0.0239 (5)	0.0336 (6)	−0.0066 (4)	0.0123 (5)	−0.0006 (4)
O2	0.0255 (5)	0.0263 (5)	0.0293 (6)	0.0004 (4)	0.0102 (4)	−0.0018 (4)
O3	0.0194 (5)	0.0234 (5)	0.0391 (6)	−0.0008 (4)	0.0051 (4)	−0.0006 (4)
O4	0.0233 (5)	0.0203 (5)	0.0306 (6)	0.0012 (4)	0.0025 (4)	0.0047 (4)
O5	0.0469 (7)	0.0399 (7)	0.0246 (6)	0.0141 (5)	0.0068 (5)	−0.0014 (5)
O6	0.0225 (5)	0.0263 (5)	0.0267 (6)	−0.0019 (4)	0.0066 (4)	0.0029 (4)
C1	0.0153 (6)	0.0199 (6)	0.0210 (7)	−0.0006 (5)	0.0017 (5)	0.0017 (5)
C2	0.0172 (6)	0.0231 (7)	0.0226 (7)	0.0010 (5)	0.0003 (5)	0.0032 (5)
C3	0.0253 (7)	0.0254 (7)	0.0215 (7)	−0.0002 (5)	−0.0029 (5)	0.0021 (6)
C4	0.0261 (7)	0.0204 (7)	0.0226 (7)	−0.0017 (5)	0.0014 (5)	0.0001 (5)
C5	0.0168 (6)	0.0193 (6)	0.0182 (6)	−0.0006 (5)	0.0011 (5)	0.0016 (5)
C6	0.0159 (6)	0.0186 (6)	0.0200 (6)	−0.0002 (5)	0.0018 (5)	0.0009 (5)
C7	0.0193 (6)	0.0190 (6)	0.0199 (7)	0.0032 (5)	0.0017 (5)	0.0003 (5)
C8	0.0229 (7)	0.0255 (7)	0.0233 (7)	0.0009 (5)	0.0027 (5)	0.0025 (6)
C9	0.0325 (8)	0.0326 (8)	0.0249 (8)	0.0052 (6)	0.0079 (6)	0.0052 (6)
C10	0.0389 (9)	0.0399 (9)	0.0188 (7)	0.0123 (7)	0.0014 (6)	0.0011 (7)
C11	0.0287 (8)	0.0418 (9)	0.0247 (8)	0.0059 (6)	−0.0048 (6)	−0.0056 (7)
C12	0.0212 (6)	0.0290 (7)	0.0247 (7)	0.0007 (5)	0.0018 (5)	−0.0032 (6)
C13	0.0199 (6)	0.0221 (7)	0.0232 (7)	0.0026 (5)	0.0037 (5)	0.0040 (5)
C14	0.0346 (8)	0.0249 (8)	0.0334 (9)	0.0059 (6)	0.0106 (6)	−0.0043 (6)
C15	0.0447 (10)	0.0349 (9)	0.0331 (9)	0.0027 (7)	0.0027 (7)	−0.0063 (7)
C16	0.0217 (6)	0.0189 (6)	0.0163 (6)	0.0005 (5)	0.0020 (5)	−0.0018 (5)
C17	0.0283 (7)	0.0228 (7)	0.0335 (8)	0.0079 (6)	0.0040 (6)	0.0057 (6)
C18	0.0586 (12)	0.0717 (14)	0.0302 (10)	0.0307 (11)	0.0024 (9)	0.0099 (9)
C19	0.0214 (7)	0.0293 (8)	0.0299 (8)	0.0048 (6)	−0.0021 (6)	0.0048 (6)

### Geometric parameters (Å, °)

**Table:**

O1—C13	1.2078 (17)	C7—C8	1.3995 (19)
O2—C13	1.3369 (17)	C8—C9	1.387 (2)
O2—C14	1.4599 (18)	C8—H8A	0.9300
O3—C16	1.2088 (16)	C9—C10	1.386 (2)
O4—C16	1.3283 (17)	C9—H9A	0.9300
O4—C17	1.4634 (17)	C10—C11	1.383 (2)
O5—C4	1.2088 (17)	C10—H10A	0.9300
O6—C2	1.4424 (17)	C11—C12	1.390 (2)
O6—H1O6	1.01 (3)	C11—H11A	0.9300
C1—C13	1.512 (2)	C12—H12A	0.9300
C1—C6	1.5442 (18)	C14—C15	1.506 (2)
C1—C2	1.5499 (19)	C14—H14A	0.9700
C1—H1A	0.9800	C14—H14B	0.9700
C2—C19	1.5220 (19)	C15—H15A	0.9600
C2—C3	1.527 (2)	C15—H15B	0.9600
C3—C4	1.505 (2)	C15—H15C	0.9600
C3—H3A	0.9700	C17—C18	1.485 (2)
C3—H3B	0.9700	C17—H17A	0.9700
C4—C5	1.5233 (19)	C17—H17B	0.9700
C5—C16	1.5168 (18)	C18—H18A	0.9600
C5—C6	1.5468 (19)	C18—H18B	0.9600
C5—H5A	0.9800	C18—H18C	0.9600
C6—C7	1.5201 (19)	C19—H19A	0.9600
C6—H6A	0.9800	C19—H19B	0.9600
C7—C12	1.3908 (19)	C19—H19C	0.9600
			
C13—O2—C14	116.77 (11)	C11—C10—C9	119.38 (14)
C16—O4—C17	117.13 (11)	C11—C10—H10A	120.3
C2—O6—H1O6	106.9 (17)	C9—C10—H10A	120.3
C13—C1—C6	108.32 (11)	C10—C11—C12	120.49 (14)
C13—C1—C2	111.74 (11)	C10—C11—H11A	119.8
C6—C1—C2	111.44 (11)	C12—C11—H11A	119.8
C13—C1—H1A	108.4	C11—C12—C7	120.63 (14)
C6—C1—H1A	108.4	C11—C12—H12A	119.7
C2—C1—H1A	108.4	C7—C12—H12A	119.7
O6—C2—C19	110.12 (11)	O1—C13—O2	123.86 (13)
O6—C2—C3	104.43 (11)	O1—C13—C1	124.38 (12)
C19—C2—C3	110.73 (12)	O2—C13—C1	111.76 (11)
O6—C2—C1	110.92 (10)	O2—C14—C15	110.74 (12)
C19—C2—C1	111.35 (11)	O2—C14—H14A	109.5
C3—C2—C1	109.08 (11)	C15—C14—H14A	109.5
C4—C3—C2	111.87 (11)	O2—C14—H14B	109.5
C4—C3—H3A	109.2	C15—C14—H14B	109.5
C2—C3—H3A	109.2	H14A—C14—H14B	108.1
C4—C3—H3B	109.2	C14—C15—H15A	109.5
C2—C3—H3B	109.2	C14—C15—H15B	109.5
H3A—C3—H3B	107.9	H15A—C15—H15B	109.5
O5—C4—C3	123.37 (13)	C14—C15—H15C	109.5
O5—C4—C5	122.13 (13)	H15A—C15—H15C	109.5
C3—C4—C5	114.48 (12)	H15B—C15—H15C	109.5
C16—C5—C4	110.02 (11)	O3—C16—O4	124.81 (12)
C16—C5—C6	109.83 (10)	O3—C16—C5	123.32 (12)
C4—C5—C6	112.23 (11)	O4—C16—C5	111.88 (11)
C16—C5—H5A	108.2	O4—C17—C18	109.29 (13)
C4—C5—H5A	108.2	O4—C17—H17A	109.8
C6—C5—H5A	108.2	C18—C17—H17A	109.8
C7—C6—C1	113.02 (11)	O4—C17—H17B	109.8
C7—C6—C5	110.25 (11)	C18—C17—H17B	109.8
C1—C6—C5	110.26 (10)	H17A—C17—H17B	108.3
C7—C6—H6A	107.7	C17—C18—H18A	109.5
C1—C6—H6A	107.7	C17—C18—H18B	109.5
C5—C6—H6A	107.7	H18A—C18—H18B	109.5
C12—C7—C8	118.50 (13)	C17—C18—H18C	109.5
C12—C7—C6	120.19 (12)	H18A—C18—H18C	109.5
C8—C7—C6	121.30 (12)	H18B—C18—H18C	109.5
C9—C8—C7	120.55 (13)	C2—C19—H19A	109.5
C9—C8—H8A	119.7	C2—C19—H19B	109.5
C7—C8—H8A	119.7	H19A—C19—H19B	109.5
C10—C9—C8	120.45 (15)	C2—C19—H19C	109.5
C10—C9—H9A	119.8	H19A—C19—H19C	109.5
C8—C9—H9A	119.8	H19B—C19—H19C	109.5
			
C13—C1—C2—O6	66.12 (14)	C1—C6—C7—C8	60.00 (16)
C6—C1—C2—O6	−55.22 (14)	C5—C6—C7—C8	−63.89 (16)
C13—C1—C2—C19	−56.89 (14)	C12—C7—C8—C9	0.4 (2)
C6—C1—C2—C19	−178.22 (11)	C6—C7—C8—C9	179.40 (13)
C13—C1—C2—C3	−179.39 (11)	C7—C8—C9—C10	−0.2 (2)
C6—C1—C2—C3	59.27 (14)	C8—C9—C10—C11	−0.2 (2)
O6—C2—C3—C4	62.27 (14)	C9—C10—C11—C12	0.3 (2)
C19—C2—C3—C4	−179.24 (12)	C10—C11—C12—C7	0.0 (2)
C1—C2—C3—C4	−56.36 (15)	C8—C7—C12—C11	−0.3 (2)
C2—C3—C4—O5	−128.63 (15)	C6—C7—C12—C11	−179.30 (13)
C2—C3—C4—C5	52.96 (16)	C14—O2—C13—O1	−8.1 (2)
O5—C4—C5—C16	9.17 (18)	C14—O2—C13—C1	170.78 (11)
C3—C4—C5—C16	−172.40 (11)	C6—C1—C13—O1	71.82 (17)
O5—C4—C5—C6	131.79 (14)	C2—C1—C13—O1	−51.30 (17)
C3—C4—C5—C6	−49.77 (15)	C6—C1—C13—O2	−107.10 (12)
C13—C1—C6—C7	56.26 (14)	C2—C1—C13—O2	129.77 (12)
C2—C1—C6—C7	179.57 (11)	C13—O2—C14—C15	−78.28 (17)
C13—C1—C6—C5	−179.85 (10)	C17—O4—C16—O3	3.5 (2)
C2—C1—C6—C5	−56.55 (14)	C17—O4—C16—C5	−176.62 (11)
C16—C5—C6—C7	−61.15 (13)	C4—C5—C16—O3	77.32 (16)
C4—C5—C6—C7	176.13 (10)	C6—C5—C16—O3	−46.70 (17)
C16—C5—C6—C1	173.38 (10)	C4—C5—C16—O4	−102.57 (13)
C4—C5—C6—C1	50.65 (14)	C6—C5—C16—O4	133.41 (12)
C1—C6—C7—C12	−121.02 (14)	C16—O4—C17—C18	104.66 (16)
C5—C6—C7—C12	115.09 (13)		

### Hydrogen-bond geometry (Å, °)

**Table:**

*D*—H···*A*	*D*—H	H···*A*	*D*···*A*	*D*—H···*A*
O6—H1O6···O1^i^	1.01 (3)	2.05 (3)	2.9958 (18)	156 (2)
C1—H1A···O3^ii^	0.98	2.44	3.323 (2)	150
C8—H8A···O3^ii^	0.93	2.60	3.493 (2)	162
C12—H12A···O2^iii^	0.93	2.56	3.468 (2)	166
C19—H19C···O6^ii^	0.96	2.55	3.396 (2)	146

Symmetry codes: (i) −*x*, −*y*+1, −*z*; (ii) *x*−1, *y*, *z*; (iii) *x*+1, *y*, *z*.
